# Robust Object Segmentation Using a Multi-Layer Laser Scanner

**DOI:** 10.3390/s141120400

**Published:** 2014-10-29

**Authors:** Beomseong Kim, Baehoon Choi, Minkyun Yoo, Hyunju Kim, Euntai Kim

**Affiliations:** 1 Electrical and Electronic Engineering Department, Yonsei University, Seoul 120-749, Korea; E-Mails: battlebs@yonsei.ac.kr (B.K.); choibae@yonsei.ac.kr (B.C.); 2 Advanced Driver Assistance System Recognition Development Team, Hyundai Motors, Gyeonggi 445-706, Korea; E-Mails: mkyoo83@hyundai.com (M.Y.); hjkim74@hyundai.com (H.K.)

**Keywords:** laser scanner, obstacle detection, segmentation, advanced driver assistance system (ADAS)

## Abstract

The major problem in an advanced driver assistance system (ADAS) is the proper use of sensor measurements and recognition of the surrounding environment. To this end, there are several types of sensors to consider, one of which is the laser scanner. In this paper, we propose a method to segment the measurement of the surrounding environment as obtained by a multi-layer laser scanner. In the segmentation, a full set of measurements is decomposed into several segments, each representing a single object. Sometimes a ghost is detected due to the ground or fog, and the ghost has to be eliminated to ensure the stability of the system. The proposed method is implemented on a real vehicle, and its performance is tested in a real-world environment. The experiments show that the proposed method demonstrates good performance in many real-life situations.

## 1. Introduction

With the recent developments in vehicular technology, advanced driver assistance system (ADAS) concept has spread rapidly; however, many problems still remain to be addressed before the field of ADASs can be widely expanded. The biggest problem in ADAS is the use of sensor measurements and recognition of the surrounding environment. To this end, several types of sensors have been considered, including radar and a visual or infrared (IR) camera. Unfortunately, however, none of the sensors are sufficient for ADAS, and each has its own shortcomings.

For example, radar returns relatively accurate distances to obstacles, but its bearing measurements are not accurate. Radar cannot recognize object classes, and also suffers from frequent false detections [1–4]. A visual camera is another ADAS tool. This type of camera returns a relatively accurate bearing to the obstacle, but its distance measurement is not reliable. The camera is capable of object recognition, but it also exhibits a high false detection rate. Thus, most of the current systems combine several sensors to compensate for the drawbacks of each sensor and to obtain reliable information about the nearby environments [5–9].

Recently, the laser scanner has received attention within the ADAS community, and it is considered to be a strong candidate for the primary sensor in ADAS [1,10]. The strong points of the laser scanner are its ability to accurately determine both near and far distances as well as the bearing to an obstacle. In addition, the object detection of a laser scanner is reliable and robust, and it can recognize object classes to some extent through determination of the contour of the surrounding environment.

Thus, unlike a camera or radar, a laser scanner can be used as the sole sensor for ADAS without being combined with other sensors. Further, if the laser scanner is combined with other sensors, it can compensate for all the drawbacks, thereby improving the recognition accuracy.

Because the range and bearing measurements of the laser scanner are sufficiently accurate, in this paper, the laser scanner is considered as a single sensor for ADAS. In this paper, the laser scanners are divided into three types according to the number of layers; single-layer, multi-layer, and three-dimensional (3D) laser scanners. A single-layer laser scanner consists of only one layer, a multi-layer laser scanner is composed more than one but fewer than eight layers, and a 3D laser scanner is composed of eight or more layers. A single-layer laser scanner can obtain 2-dimensional (2D) information, a 3D laser scanner can get 3D information, and a multi-layer laser scanner can get limited 3D information. In general, the information from a laser scanner is proportional to the number of layers, but a 3D layer scanner is expensive and difficult to install on the vehicle. However, single-layer and multi-layer laser scanners can be implemented inside a vehicle's body [11]. Therefore, the 3D layer scanner is not yet suitable for ADAS, and the multi-layer laser scanner is currently more suitable for ADAS.

The remainder of this paper is organized as follows: in Section 2, an outline of the obstacle recognition system using a laser scanner is described. Related works are described in Section 3. The segmentation for a multi-layer laser scanner and the ghost elimination are explained in Section 4. The proposed system is installed on a vehicle and is applied to actual urban road navigation. The experimental results are presented in Section 5. The discussion about robustness is described in Section 6. Finally, some conclusions are drawn in Section 7.

## 2. System Outline

**Figure 1.**
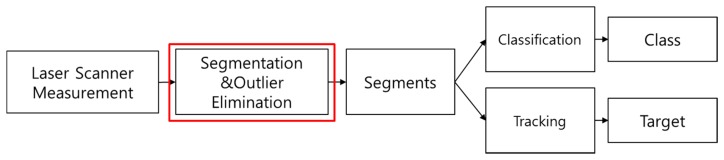
System Outline.

The system aims to detect obstacles through the processes of segmentation, classification, and tracking. Figure 1 shows the outline of the system developed in this paper.

In the segmentation step, the proposed system receives a full scan (a set) of measurement points from a multi-layer laser scanner and decomposes the set into several segments, each of which corresponds to an object. In the segmentation step, the outliers are removed to avoid performance degradation. In the classification step, the segment features are computed and classified [12–14]. In the tracking step, the location and velocity of the segment are estimated over time. Segmentation is the essential step to execute classification or tracking [15–18]. In this paper, we focus on the methods for segmentation and outlier elimination.

## 3. Related Works

**Figure 2.**
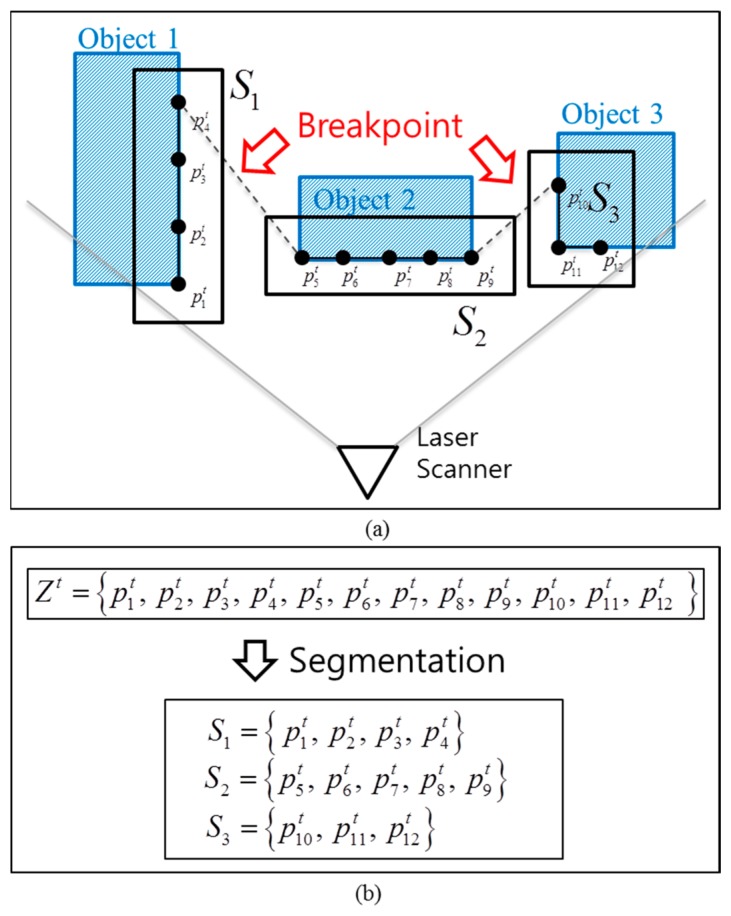
Segmentation by a single-layer laser scanner, (**a**) example of laser scanner measurement; (**b**) result of segmentation.

A laser scanner detects the closest obstacle for a bearing angle and returns the angle-wise distance to the obstacle. The output of the laser scanner can be modeled by the following set of pairs:

(1) $$Z^{t}=\left\{p_{1}^{t},p_{2}^{t},p_{3}^{t},\cdots ,p_{N}^{t}\right\}$$

(2) $$p_{i}^{t}=\left(r_{i}^{t},\theta_{i}^{t}\right)\;\text{for}\;i=1,\ldots ,N$$

(3) $$\theta_{i}^{t}>\theta_{i-1}^{t}\;\text{for}\;i=2,\ldots ,N$$

where *N* denotes the number of scanner measurement points as shown in Figure 2; the superscript *t* denotes the measurement time; and $r_{i}^{t}$ and $\theta_{i}^{t}$ denote the distance and bearing to the obstacles, respectively. Equation (3) refers to a property of the laser scanner, and it implies that the scanner scans the environment from left to right. As stated in Section 2, segmentation is the first step for the object detection by the laser scanner. A scan of the measurements, as given in Equations (1) and (2), is decomposed into several groups called segments, as shown in Figure 2.

In general, the segmentation methods can be classified into two kinds: the geometric shape method and the breakpoint detection (BD) method. The first method assumes the geometric shapes of the segments, such as a line or an edge, and decomposes the scanner measurements into the predetermined shapes [19,20]. The BD method decomposes the scanner measurements into segments based on the Euclidean distance between the consecutive points $p_{i}^{t}$ and $p_{i-1}^{t}$ [21] or using a Kalman filter [22]. Methods using BD based on the distances between consecutive points are the most popular and are widely used for laser segmentation [23–27].

In the distance-based BD, if the distance between two consecutive points is greater than a threshold *D_thd_*, two points are likely to belong to different objects and a breakpoint is selected between the two points as shown in Figure 2a.

In Figure 2, the laser scanner returns 12 data points ( $p_{1}^{t}-p_{12}^{t}$) and the segment *S_n_* originates from the *n* th object (*n* = 1,2,3). The performance of the BD segmentation depends on the choice of a threshold *D_thd_*, and several methods have been developed for the selection of *D_thd_*. In [23], the threshold *D_thd_* is determined by:

(4) $$D_{\text{thd}}=C_{0}+C_{1}\min\;\left\{r_{i}^{t},r_{i-1}^{t}\right\}$$

where $C_{1}=\sqrt{2\left(1-\cos\left(\theta_{i}^{t}-\theta_{i-1}^{t}\right)\right)}$ and *C*_0_ denotes the sensor noise. In [24], Lee *et al.*, employed Equation (5) to select the break points in [17].

(5) $$D_{\text{thd}}=\left|\frac{r_{i}^{t}-r_{i-1}^{t}}{r_{i}^{t}+r_{i-1}^{t}}\right|$$

**Figure 3.**
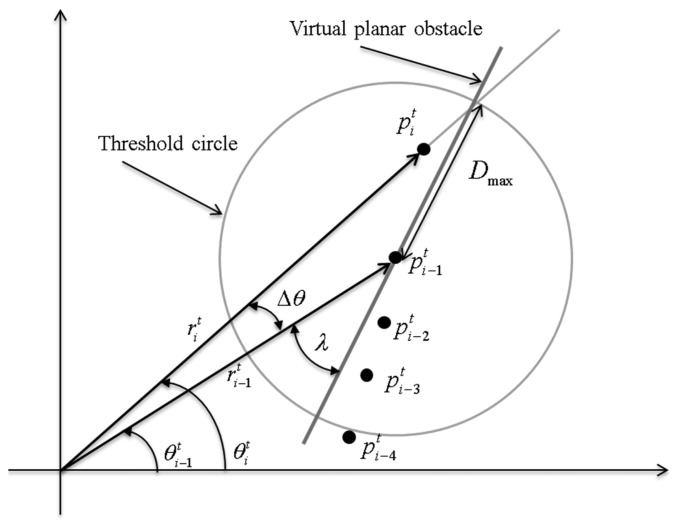
Adaptive Breakpoint Detector (ABD).

Borges *et al.*, recently proposed the adaptive breakpoint detector (ABD) in [25]. In the ABD, the threshold *D_thd_* is determined by:

(6) $$D_{\text{thd}}=r_{i-1}^{t}\cdot \frac{\sin\left(\Delta \theta \right)}{\sin\left(\lambda -\Delta \theta \right)}+3\sigma_{r}$$

which adaptively depends on $r_{i-1}^{t}$ and Δ*θ* as shown in Figure 3. In Equation (6), $\Delta \theta =\theta_{i}^{t}-\theta_{i-1}^{t}$, where *λ* is chosen on the basis of user experience, and *σ_r_* is the sensor noise associated with *r*.

All of the previous segmentation methods, however, were based on a single-layer laser scanner, and to our knowledge, no research has been reported regarding the segmentation for a multi-layer laser scanner. This is one of the contributions of this paper.

## 4. Segmentation for Multi-Layer Laser Scanner

### 4.1. ABD Segmentation for Multi-Layer Laser Scanner

**Figure 4.**
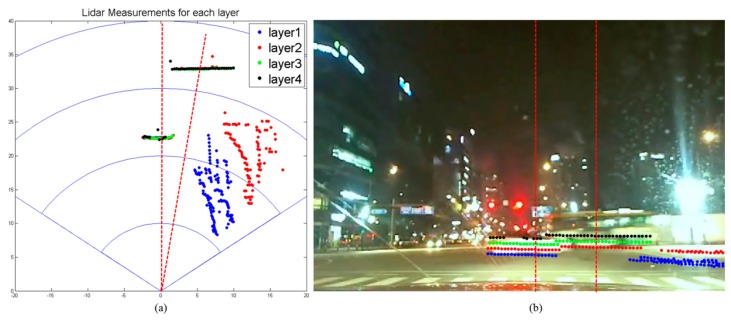
Multi-layer laser scanner (**a**) scan data and (**b**) corresponding image.

A multi-layer laser scanner has multiple layers and returns the measurement points as shown in Figure 4.

Figure 4a shows the laser scanner measurements depicted on the *x-y* plane, and Figure 4b shows the scanner measurements superimposed on the camera image after calibration [28]. In the figure, the information of the different layers is represented by different colors. In the multi-layer laser, each data point $p_{i}^{t}$ is not a pair but a triplet consisting of the distance $r_{i}^{t}$ and bearing $\theta_{i}^{t}$ to the obstacle and the layer information $l_{i}^{t}$. The output of the multi-layer laser scanner is modeled by:

(7) $$\begin{array}{cc} p_{i}^{t}=\left(r_{i}^{t},\theta_{i}^{t},l_{i}^{t}\right) & \text{for}\; \end{array}i=1,\ldots ,N$$

(8) $$\begin{array}{cc} \theta_{i}^{t}\geq \theta_{i-1}^{t} & \text{for}\;i=2,\ldots ,N \end{array}$$

(9) $$\begin{array}{cc} l_{i}^{t}\geq l_{i-1}^{t} & \text{where}\; \end{array}\theta_{i}^{t}=\theta_{i-1}^{t}$$

where a four layer laser scanner, the IBEO LUX2010 [29], is used, and:

(10) $$l_{i}^{t}\in \left\{1,2,3,4\right\}$$

Equations (8) and (9) refer to the property that the scanner scans the laser from left to right and from bottom to top, respectively. The left points are measured before the right points and at the same $\theta_{i}^{t}$, and the lower layers are measured before the upper layers. The direct application of a standard ABD to multi-layer segmentation would lead to the loss of layer information and would lead to inefficient segmentation.

**Figure 5.**
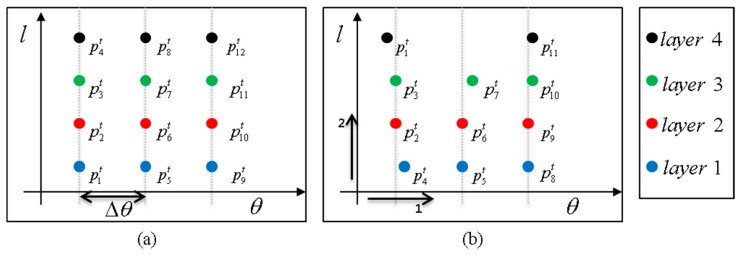
Scan points of the multi-layer laser scanner on the *θ - l* plane. (**a**) ideal output (**b**) actual output.

In order to develop a new segmentation method for the multi-layer scanner, two important properties of the multi-layer laser scanner should be considered:

(1) Two points at the same bearing but on different layers can belong to different objects. An example is the situation given in Figure 4. In the figure, both a sedan and a bus lie in the same bearing, but the sedan is closer to the scanner than the bus. From the box bounded in red dotted lines in Figure 4b, the points in the lower three layers belong to the sedan, but the points in the top layer belong to the bus. Thus, it must be determined whether or not two data points, $p_{i}^{t}$ and $p_{j}^{t}$, with consecutive bearings and consecutive layers belong to the same object.
(2) The measurement sets are not complete, and there are many vacancies in the *θ - l* plane. When the scanner input is plotted on the *θ - l* plane, the ideal output will look like a grid as in Figure 5a, but the actual output appears as in Figure 5b. Thus, the grid-type segmentation using a nested for-loop cannot be used.

A layer-wise independent segmentation process can be considered; however, in our experience, this method does not work well and requires multi-layer segmentation. In multi-layer laser segmentation, we will say that two points $p_{i}^{t}$ and $p_{j}^{t}$ are *connected* if they belong to the same object. Unlike single-layer scanner segmentation, we consider not only the connectivity between the points with consecutive bearings, but also the connectivity between the points with consecutive layers. The foremost requirement in multi-layer segmentation is that the algorithm should operate in a single scan with the running time *O*(*N*), and the algorithm must not trace back to the old previous points, making it *O*(*N*^2^) or higher, where *N* is the number of measurement points.

**Figure 6.**
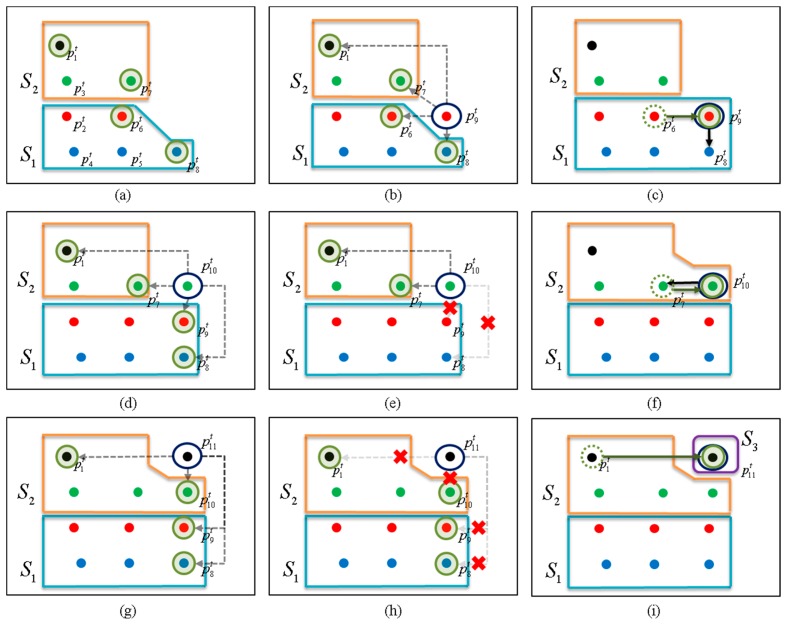
ABD segmentation for multi-layer laser scanner.

In this paper, an *O*(*N*) fast segmentation method is presented. When each data point $p_{i}^{t}$ is given, a *candidate set* 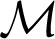
*_i_* composed of previous data points $p_{j}^{t}$ (*j*<*i*) is built, and the connectivity of the point $p_{i}^{t}$ is tested only with the elements in 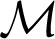
*_i_*, thereby implementing an *O*(*N*) implementation. In our segmentation method, the candidate set 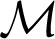
*_i_* consists of the newest data points in each layer. Therefore, the maximum size of the candidate set 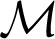
*_i_* is four in this case. Figure 6 illustrates the ABD segmentation process.

In Figure 6, we assume that eight data points ( $p_{1}^{t}-p_{8}^{t}$) have already been received. $p_{2}^{t}$, $p_{4}^{t}$, $p_{5}^{t}$, $p_{6}^{t}$, and $p_{8}^{t}$ belong to the segment *S*_1_, and $p_{1}^{t}$, $p_{3}^{t}$, and $p_{7}^{t}$ belong to *S*_2_, as in Figure 6a. This situation occurs as in Figure 4, in which two vehicles are in the same direction but at the different distances. The candidate set 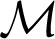
_9_ is composed of $p_{1}^{t}$, $p_{6}^{t}$, $p_{7}^{t}$ and $p_{8}^{t}$. The four points in 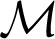
_9_ are the newest points in each layer at this time and the points in the set will be checked when a new point $p_{9}^{t}$ arrives. The points in 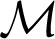
_9_ are indicated by the green circles in Figure 6a.

In Figure 6b, $p_{9}^{t}$ is presented, and its connectivity with the elements in 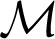
_9_ is determined in turn from the first to the fourth layers (in order of $p_{8}^{t}$, $p_{6}^{t}$, $p_{7}^{t}$, and $p_{1}^{t}$) with using an ABD. Here, we assume that the data point $p_{9}^{t}$ is assigned to the first segment as in Figure 6c. For example, if $\left\|p_{9}^{t}-p_{8}^{t}\right\|\leq D_{\text{thd}}$, then $p_{9}^{t}$ is assigned to the segment of $p_{8}^{t}$, which is *S*_1_, and further connectivity determinations with $p_{6}^{t}$, $p_{7}^{t}$, and $p_{1}^{t}$ are cancelled. Figure 6d-f show the segmentation of $p_{10}^{t}$. First, 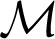
_10_ is computed by:

(11) $$\begin{array}{ll} M_{10} & =M_{9}\cup \left\{p_{9}^{t}\right\}-\left\{p_{6}^{t}\right\}\\ & =\left\{p_{1}^{t},p_{7}^{t},p_{8}^{t},p_{9}^{t}\right\} \end{array}$$

in Figure 6d, where ∪ and − denote set union and subtraction, respectively. As before, the connectivity of $p_{10}^{t}$ with $p_{8}^{t}$, $p_{9}^{t}$, $p_{7}^{t}$, and $p_{1}^{t}$ is tested in turn (in order of $p_{8}^{t}$, $p_{9}^{t}$, $p_{7}^{t}$, and $p_{1}^{t}$). If $\left\|p_{10}^{t}-p_{8}^{t}\right\|>D_{\text{thd}}$ and $\left\|p_{10}^{t}-p_{9}^{t}\right\|>D_{\text{thd}}$ but $\left\|p_{10}^{t}-p_{7}^{t}\right\|\leq D_{\text{thd}}$ as in Figure 6e, then $p_{10}^{t}$ is assigned to the segment of $p_{7}^{t}$, which is *S*_2_, and further connectivity determination with $p_{1}^{t}$ is cancelled, as in Figure 6f.

In a similar way, $p_{11}^{t}$ is segmented in Figure 6g–i. As before, 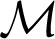
_11_ is updated by:

(12) $$\begin{array}{ll} M_{11} & =M_{10}\cup \left\{p_{10}^{t}\right\}-\left\{p_{7}^{t}\right\}\\ & =\left\{p_{1}^{t},p_{8}^{t},p_{9}^{t},p_{10}^{t}\right\} \end{array}$$

as in Figure 6g. The connectivity of $p_{11}^{t}$ is tested with the elements $p_{8}^{t}$, $p_{9}^{t}$, $p_{10}^{t}$ and $p_{1}^{t}$ in 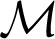
_11_ in turn. If all of the distances between $p_{11}^{t}$ and the elements in 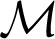
_11_ are larger than *D_thd_* by $\left\|p_{11}^{t}-p_{j}^{t}\right\|>D_{\text{thd}}$ (*j* = 1,8,9,10), as in Figure 6h, then a new segment *S*_3_ is created, and $p_{11}^{t}$ is assigned to *S*_3_ (Figure 6i).

Table 1 shows the proposed segmentation algorithm for the multi-layer laser scanner. In the table, *N_seg_* denotes the number of segments, and *N* and *L* denote the number of data points and the number of layers, respectively. *S*_1_ and 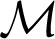
_1_ are initialized with empty sets, and the segmentation proceeds from $p_{1}^{t}$ to $p_{N}^{t}.$

In the *i* th iteration, the connectivity of $p_{i}^{t}$ with the elements in 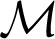
*_i_* is tested from the bottom layer to the top layer. The connectivity exist when the distance between $p_{i}^{t}$ and $p_{j}^{t}$ is smaller than threshold, *D_thd_*. $p_{j}^{t}$ is one of elements in 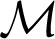
*_i_* and *D_thd_* is calculated using an ABD. If the $p_{j}^{t}$ is the first matching connected point, the $p_{i}^{t}$ is assigned to *S_n_*. *S_n_* is the segment that contains the first matching point $p_{j}^{t}.$

If $p_{i}^{t}$ is *not* close enough to any elements in 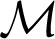
*_i_* and $p_{i}^{t}$ is not connected to any segment, then it means that $p_{i}^{t}$ belongs to a new segment and we increase *N_seg_* by one. At the end of each iteration, we update the candidate set 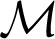
*_i_*_+1_ using 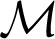
*_i_* and $p_{i}^{t}.$

### 4.2. Robust Segmentation through Ghost Elimination

**Figure 7.**
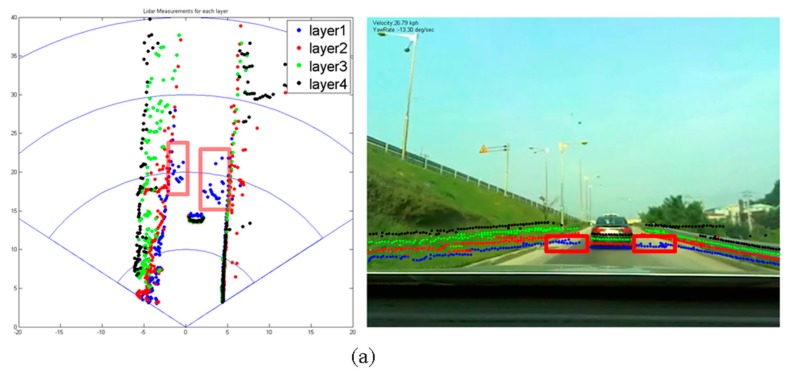

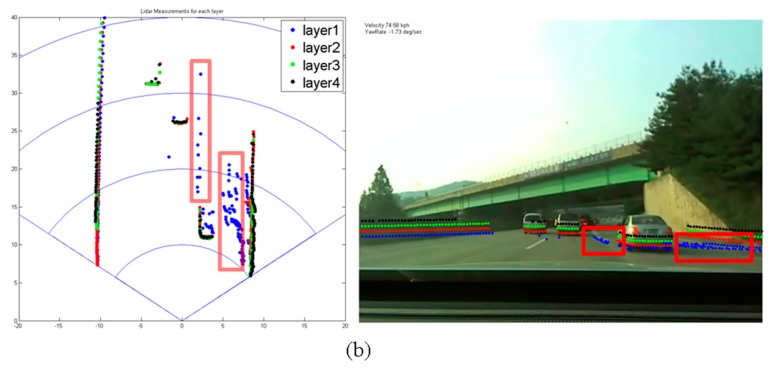
Ghost detection caused by reflection from the ground surface, (**a**) uphill road (**b**) flat road.

**Figure 8.**
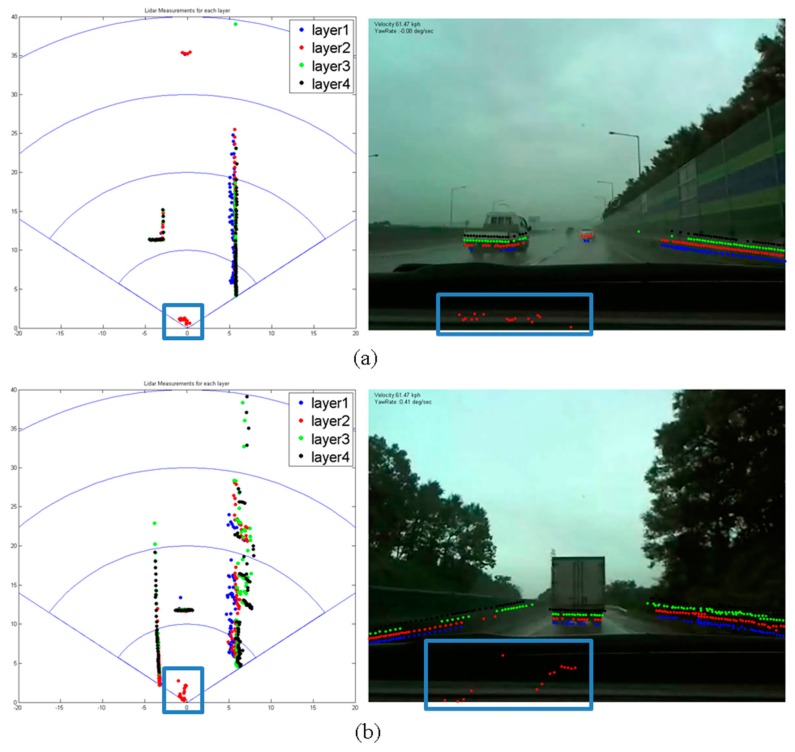
A ghost caused by moisture. (**a**) rainy weather (**b**) foggy weather.

When the above ABD segmentation is applied to actual roads, ghost segments are sometimes detected. Here, a ghost segment refers to a segment that does not actually exist but that is detected by the laser scanner. Figures 7 and 8 show examples of ghost segments. These ghosts pose a serious risk to safe driving. Most ghost segments are caused by (1) laser reflections from the ground surface; or (2) lights from vehicles or fog. The false determination of a ghost segment seriously degrades the subsequent object classification performance.

Ghosts that are caused by reflections from the ground surface often occur when vehicles travel over bumpy roads or when they go up- or downhill. Ghost segments are detected on only one layer, usually the first layer, within a 40-m distance of the scanner, and experience rapid change, appearing and disappearing and changing shape. Ghosts that are caused by headlights or tail lights or by nearby fog are also detected on only a single layer within a 20-m distance of the scanner, do not have a uniform shape, and are detected intermittently.

The two kinds of ghosts both exist only on a single layer and are detected within a short distance. Thus, our robust segmentation method is developed by considering the first property and applying it within a limited 40-m distance around the vehicle. For distances greater than 40 m, the ABD segmentation method explained in Section 4.1 is used.

The robust segmentation method is similar to the ABD segmentation given in Section 4.1, with two main differences. The first difference is that when a point, $p_{i}^{t}$, is presented in robust segmentation, the point is not segmented with a point on the same layer in 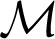
*_i_*. The reason for this is that ghost points tend to gather only on a single layer. By not combining a new point with the points on the same layer, ghost points do not build a meaningful segment and thus are not considered. The second difference is that the candidate set 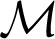
*_i_* consists of not one but two of the newest points in each other layer. To prevent an object from being divided due to a ghost, we determine the connectivity of up to two of the newest points in each other layer.

**Figure 9.**
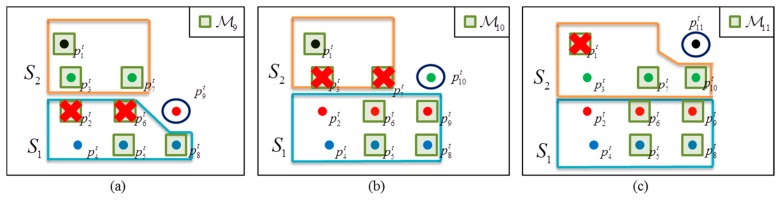
Robust segmentation for multi-layer laser scanner.

Thus, 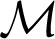
*_i_* in robust segmentation can have up to eight (2×4) elements. Figure 9 illustrates the robust segmentation process. When a new point, $p_{9}^{t}$, is presented, as in Figure 9a, a candidate set, $M_{9}=\left\{p_{1}^{t},p_{2}^{t},p_{3}^{t},p_{5}^{t},p_{6}^{t},p_{7}^{t},p_{8}^{t}\right\}$, is given. When testing the connectivity of $p_{9}^{t}$ with other points in 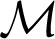
_9_, we skip the test with $p_{2}^{t}$, and $p_{6}^{t}$ because they are on the same layer as $p_{9}^{t}$. Thus, the connectivities of $p_{9}^{t}$ are tested only with the five points { $p_{1}^{t}$, $p_{3}^{t}$, $p_{5}^{t}$, $p_{7}^{t}$, $p_{8}^{t}$ } in 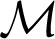
_9_, as shown in Figure 9a. Figures 9b and c demonstrate the computation of 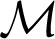
_10_ and 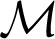
_11_ and the robust segmentation process when $p_{10}^{t}$ and $p_{11}^{t}$ are given, respectively.

The problem with this approach is that if an obstacle is very shallow and is detected only on a single layer, it may not be detected. However, this is rarely the case due to the sufficiently small angular resolution of the multi-layer laser scanner. In the case of the IBEO LUX2010, the vertical and horizontal angular resolutions are 0.8° and 0.125°, respectively, allowing an obstacle 20 m or more from the vehicle and larger than 0.56 m to be detected on more than two layers. If the obstacle is larger than 0.26 m, it will return more than six points.

Table 2 shows the pseudo-code of the robust segmentation. In lines 4-14, when a new point, $p_{i}^{t}$, is within 40 m of the scanner, the connectivity determination with the point on the same layer is skipped, as Figure 9. In lines 15–24, when a new point is far from the scanner, its connectivity with the point on the same layer is determined as the ABD segmentation. The processes of making new segment and updating candidate set, 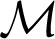
*_i_*_+l_, is same as the processes of ABD segmentation. End of this algorithm, small segments are eliminated. The small segments mean the number of point is smaller than *N*_min_, and *N*_min_ denotes the minimum number of points required for an object.

## 5. Experiment

**Figure 10.**
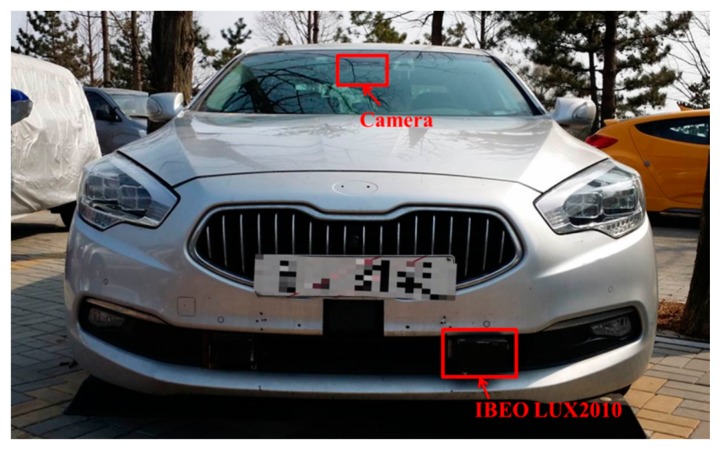
Vehicle and laser scanner for the experiment.

In this experiment, an IBEO LUX2010 multi-layer laser scanner and a camera are installed on a Kia K900 as shown in Figure 10. As previously stated, the LUX2010 has a total of four layers, and its horizontal and vertical resolutions are 0.125° and 0.8°, respectively. The camera is used to obtain the ground truth of the environment.

**Figure 11.**
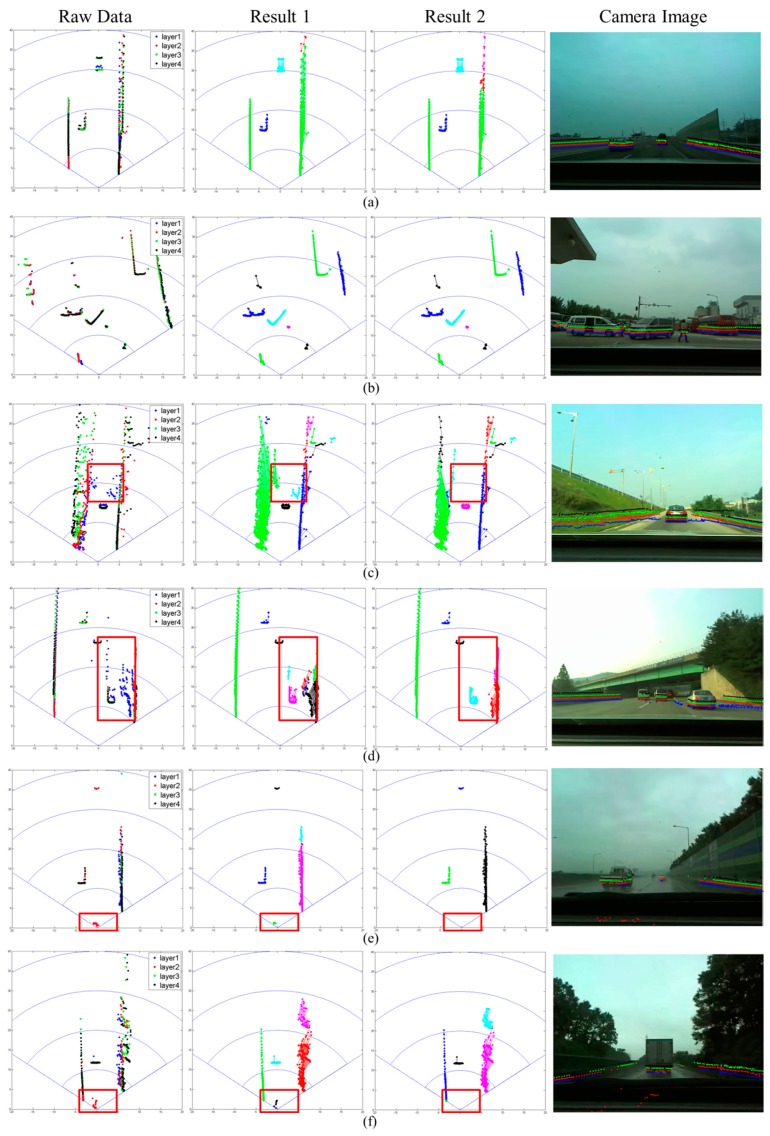
Segmentation results: (**a**) vehicle; (**b**) pedestrian; (**c**) ghost in uphill road; (**d**) ghost in flat road; (**e**) ghost created by rain; and (**f**) ghost created by fog. Result 1 (second column)—ABD segmentation; Result 2—robust segmentation.

Figure 11 shows the segmentation results for six different scenarios. The first column shows the raw measurements from the IBEO scanner, and the second and third columns show the ABD and robust segmentation results, respectively. The fourth column contains the corresponding camera image with the scanner measurements superimposed.

Figure 11a and b show the results when the road is flat and ghosts are not detected. Only vehicles appear in Figure 11a, while both vehicles and pedestrians appear in Figure 11b. In the two scenarios, ghosts are not observed, and it can be seen that the ABD and robust segmentations produce the same results.

Figure 11c and d show the results when a ghost is detected that is created by the surface. In the figures, the dots in the red box are the ghost, detected by the bottom layer laser, which is indicated in blue. When the ABD segmentation method is applied (second column), the ghost forms an outlier segment and appears to be an obstacle. When the robust segmentation method is applied (third column), however, the ghost is successfully removed, leaving only the segments from the preceding vehicles.

Figure 11e and f show the results in rainy, foggy test conditions. As in Figure 11c and d, the dots in the red box are detected by the second-layer laser, and appear to result from fog. When the ABD segmentation method is applied (second column), the ghost survives and could activate the brake system, which can lead to an accident. When the robust segmentation method is applied (third column), however, the ghost is successfully removed. For quantitative analysis, we gather samples from four scenarios as in Table 3 and apply the ABD and robust segmentation methods.

All the samples are clipped manually from the IBEO scan. In Tables 4, 5, 6, and 7, the results of the ghost elimination are described. The value of *λ* in Equation (6) is changed from 10° to 15°. The experiments are conducted in uphill road, flat road, rainy weather, and foggy weather conditions and their results are shown in Tables 4, 5, 6, and 7, respectively.

In the tables, the ABD and robust segmentation methods are compared in terms of (1) ghost elimination ratio; (2) inlier survival ratio and (3) computation time. Here, the ghost elimination ratio and the inlier survival ratio are defined as:

(13) $$\text{Ghost elimination ratio}=\frac{\text{The number of eliminated ghosts}}{\text{The number of ghosts}}\times 100$$

(14) $$\text{Inlier survival ratio}=\frac{\text{The number of survived inliers}}{\text{The number of inliers}}\times 100$$

From the tables, the proposed robust method outperforms the ABD in all cases with the similar computation time.

## 6. Discussion

Obviously, the goal is to remove as many ghosts as possible while maintaining as many inliers as possible and, thus to keep both ratios high. It can be seen from Tables 4, 5, 6, and 7 that the results of robust segmentation are better than those of ABD segmentation in every condition. In particular, the proposed robust method demonstrates more than 95% of the ghost elimination ratio in a robust manner regardless of the weather or the road. The ABD, however, demonstrates 17% to 65% of the ghost elimination ratio depending on the weather or the road. When it rains or the car goes uphill and, thus, ghosts frequently occur, ABD fails in eliminating the ghosts but the robust method removes most of the ghosts well. Interestingly, the ABD also performs well in the foggy weather and the reason is that the ghosts are detected intermittently in the foggy weather and they tend not to form a segment. Further, the ghost elimination ratio is not much affected by the value of *λ*. The reason might be that the ghosts are very close to the sensors and they are far enough from the other obstacles.

The inlier survival ratio is also an important factor because if the inlier is accidently removed by the algorithm, it will lead to a serious accident. The result of the inlier survival ratio is also shown in Tables 3, 4, 5, and 6. It can be seen that both of the segmentation methods have sufficiently high inlier survival ratios and the both algorithms do not accidently remove the important measurement points.

The ABD and robust segmentation methods are also compared in terms of computation time. The computation time in Tables 4, 5, 6, and 7 is obtained by computing the average over 100 frames. It can be seen that the robust method takes slightly longer time than the ABD but the extra time is not much. The reason is that the ghost tends to form a number of small segment and the elimination of them takes some time.

## 7. Conclusions

In this paper, a new object segmentation method for a multi-layer laser scanner has been proposed. For robust segmentation, efficient connectivity algorithms were developed and implemented with *O*(*N*) complexity. The proposed method was installed on an actual vehicle, and its performance was tested using real urban scenarios. It was demonstrated that the proposed system works well, even under complex urban road conditions.

## Figures and Tables

**Table 1.** ABD segmentation for multi-layer laser scanner.

*S* = *ABD* _ *Segmentation _ for* _ *multi* _ *laayer* _ *laser* _ *sacanner*(*Z_t_*)

1 *S*_1_ ← Ø, 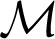 _1_ ← Ø, *N_seg_* ← 0
2 ***for*** *i* = 1 *to N* ***do***
3 *Select* $p_{i}^{t}=\left(r_{i}^{t},\theta_{i}^{t},l_{i}^{t}\right)$
4 ***for*** *all* $p_{j}^{t}$ ∈ 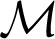 *_i_* ***do*** ⫽ 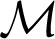 *_i_* is candidate set
5 *Select* $p_{j}^{t}=\left(r_{j}^{t},\theta_{j}^{t},l_{j}^{t}\right)$
6 *Calculate D_thd_* using ( $p_{i}^{t}$, $p_{j}^{t}$) *by ABD^a^*
7 ***if*** $\left\|p_{i}^{t}-p_{j}^{t}\right\|\leq D_{\text{thd}}$ ***then*** ⫽ check the connectivity
8 $S_{n}\leftarrow S_{n}\cup \left\{p_{i}^{t}\right\}$ *where* $p_{j}^{t}$ ∈ *S_n_* ⫽ $p_{i}^{t}$ is added to the segment *S_n_*
9 ***break***
10 ***endif***
11 ***endfor***
12 ***if*** $p_{i}^{t}\notin \overset{N_{\text{seg}}}{\underset{n=1}{\cup }}S_{n}$ ***then*** ⫽ if $p_{i}^{t}$ is not connected any other point in 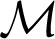 *_i_*
13 *N_seg_* ← *N_seg_* + 1
14 $S_{N_{\text{seg}}}\leftarrow \left\{p_{i}^{t}\right\}$ ⫽ $p_{i}^{t}$ belong to new segment, *S_Nseg_*
15 ***endif***
16 *Update* 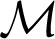 *_i+_*_1_ *from* ( 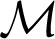 *_i_*, $p_{i}^{t}$)
17 ***endfor***

a ABD is the adaptive breakpoint detector.

**Table 2.** Pseudo-Code of Robust Segmentation through Ghost Elimination.

*S* = *Robust* _ *Segmentation*(*Z_t_*)

1 *S*_1_ ← Ø, 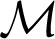 _1_← Ø, *N_seg_*← 0
2 ***for*** *i* = 1 *to N* ***do***
3 *Select* $p_{i}^{t}=\left(r_{i}^{t},\theta_{i}^{t},l_{i}^{t}\right)$
4 ***if*** ( $r_{i}^{t}\leq R_{\text{thd}}$) ***then*** ⫽ $p_{i}^{t}$ in a close area
5 ***for*** *all* $p_{j}^{t}$ ∈ 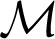 *_i_* ***do*** ⫽ 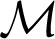 *_i_* is candidate set
6 *Select* $p_{j}^{t}=\left(r_{j}^{t},\theta_{j}^{t},l_{j}^{t}\right)$
7 ***if*** ( $l_{j}^{t}\neq l_{i}^{t}$) ***then*** ⫽ skip $p_{j}^{t}$ on same layer
8 *Calculate D_thd_* using ( $p_{i}^{t}$, $p_{j}^{t}$) *by ABD^a^*
9 ***if*** $\left\|p_{i}^{t}-p_{j}^{t}\right\|\leq D_{\text{thd}}$ ***then*** ⫽ check the connectivity
10 $S_{n}\leftarrow S_{n}\cup \left\{p_{i}^{t}\right\}$ *where* $p_{j}^{t}$ ∈ *S_n_* ⫽ $p_{i}^{t}$ is added to the segment *S_n_*
11 ***break***
12 ***endif***
13 ***endif***
14 ***endfor***
15 *else ⫽* $p_{i}^{t}$ in a far area
16 ***for*** *all* $p_{j}^{t}$ ∈ 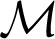 *_i_* ***do*** ⫽ 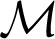 *_i_* is candidate set
17 *Select* $p_{j}^{t}=\left(r_{j}^{t},\theta_{j}^{t},l_{j}^{t}\right)$
18 *Calculate D_thd_* using ( $p_{i}^{t}$, $p_{j}^{t}$) *by ABD^a^*
19 ***if*** $\left\|p_{i}^{t}-p_{j}^{t}\right\|\leq D_{\text{thd}}$ ***then*** ⫽ check the connectivity
20 $S_{n}\leftarrow S_{n}\cup \left\{p_{i}^{t}\right\}$ *where* $p_{j}^{t}$ ∈ *S_n_* ∈ $p_{i}^{t}$ is added to the segment *S_n_*
21 ***break***
22 ***endif***
23 ***endfor***
24 ***endif***
25 ***if*** $p_{i}^{t}\notin \overset{N_{\text{seg}}}{\underset{n=1}{\cup }}S_{n}$ ***then*** ⫽ if $p_{i}^{t}$ is not connected any other point in 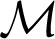 *_i_*
26 *N_seg_ ← N_seg_* + 1
27 $S_{N_{\text{seg}}}\leftarrow \left\{p_{i}^{t}\right\}$ ⫽ $p_{i}^{t}$ belong to new segment, *S_Nseg_*
28 ***endif***
29 *Update* 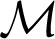 *_i_*_+1_ *from* ( 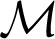 *_i_*, $p_{i}^{t}$)
30 ***endfor***
31 *Eliminate small segments in S* ⫽ *S* is the set of all segments *S_n_*

a ABD is the adaptive breakpoint detector.

**Table 3.** The number of ghosts, inliers, total measurement.

**Circumstance**	**Ghost**	**Inlier**	**Total**
Uphill road	4634	33,183	37,817
Plat road	1278	40,792	42,070
Rainy weather	2146	26,335	28,481
Foggy weather	1511	36,964	38,475

**Table 4.** Results of ghost elimination for uphill road.

*λ*	**ABD Segmentation**			**Robust Segmentation**		
	
	**Ghost Elimination Ratio (%)**	**Inlier Survival Ratio (%)**	**Computation Time (ms)**	**Ghost Elimination Ratio (%)**	**Inlier Survival Ratio (%)**	**Computation Time (ms)**
10	19.033	99.702	42.057	98.425	98.333	47.676
11	18.494	99.735	44.409	98.144	98.379	51.036
12	18.062	99.756	45.243	97.820	98.457	51.829
13	17.846	99.765	45.832	97.518	98.484	52.594
14	17.717	99.765	44.417	97.195	98.505	50.557
15	17.479	99.792	43.991	97.065	98.550	50.455

**Table 5.** Results of ghost elimination for flat road.

*λ*	**ABD Segmentation**			**Robust Segmentation**		
	
	**Ghost Elimination Ratio (%)**	**Inlier Survival Ratio (%)**	**Computation Time (ms)**	**Ghost Elimination Ratio (%)**	**Inlier Survival Ratio (%)**	**Computation Time (ms)**
10	42.097	99.980	30.193	98.513	99.909	31.147
11	41.549	99.983	29.701	98.279	99.909	30.669
12	40.141	99.983	29.386	98.122	99.909	30.413
13	38.654	99.983	29.173	97.887	99.909	30.252
14	38.419	99.983	29.117	97.731	99.909	30.111
15	37.637	99.983	29.787	97.574	99.909	31.369

**Table 6.** Results of ghost elimination in rainy weather.

*λ*	**ABD Segmentation**			**Robust Segmentation**		
	
	**Ghost Elimination Ratio (%)**	**Inlier Survival Ratio (%)**	**Computation Time (ms)**	**Ghost Elimination Ratio (%)**	**Inlier Survival Ratio (%)**	**Computation Time (ms)**
10	34.669	99.992	11.105	94.548	99.951	11.445
11	34.669	99.992	11.157	94.548	99.958	11.425
12	34.669	99.992	12.133	94.548	99.962	12.496
13	34.669	99.992	12.270	94.548	99.962	12.554
14	34.669	99.992	12.592	94.548	99.962	12.883
15	34.669	99.992	12.885	94.548	99.966	13.208

**Table 7.** Results of ghost elimination in foggy weather.

*λ*	**ABD Segmentation**			**Robust Segmentation**		
	
	**Ghost Elimination Ratio (%)**	**Inlier Survival Ratio (%)**	**Computation Time (ms)**	**Ghost Elimination Ratio (%)**	**Inlier Survival Ratio (%)**	**Computation Time (ms)**
10	63.269	99.946	21.019	97.088	99.221	23.411
11	63.203	99.946	20.161	97.022	99.261	22.484
12	63.137	99.951	20.711	96.956	99.294	23.019
13	63.071	99.954	20.983	96.889	99.294	23.375
14	63.071	99.957	21.439	96.889	99.321	23.961
15	62.674	99.959	22.869	96.823	99.359	25.473
